# Inferring Genome-Wide Recombination Landscapes from Advanced Intercross Lines: Application to Yeast Crosses

**DOI:** 10.1371/journal.pone.0062266

**Published:** 2013-05-02

**Authors:** Christopher J. R. Illingworth, Leopold Parts, Anders Bergström, Gianni Liti, Ville Mustonen

**Affiliations:** 1 Wellcome Trust Sanger Institute, Hinxton, Cambridge, United Kingdom; 2 Donnelly Centre for Cellular and Biomolecular Research, University of Toronto, Toronto, Ontario, Canada; 3 Institute of Research on Cancer and Ageing of Nice, Université de Nice Sophia Antipolis, Nice, France; National Cancer Institute, United States of America

## Abstract

Accurate estimates of recombination rates are of great importance for understanding evolution. In an experimental genetic cross, recombination breaks apart and rejoins genetic material, such that the genomes of the resulting isolates are comprised of distinct blocks of differing parental origin. We here describe a method exploiting this fact to infer genome-wide recombination profiles from sequenced isolates from an advanced intercross line (AIL). We verified the accuracy of the method against simulated data. Next, we sequenced 192 isolates from a twelve-generation cross between West African and North American yeast *Saccharomyces cerevisiae* strains and inferred the underlying recombination landscape at a fine genomic resolution (mean segregating site distance 0.22 kb). Comparison was made with landscapes inferred for a similar cross between four yeast strains, and with a previous single-generation, intra-strain cross (Mancera et al., Nature 2008). Moderate congruence was identified between landscapes (correlation 0.58–0.77 at 5 kb resolution), albeit with variance between mean genome-wide recombination rates. The multiple generations of mating undergone in the AILs gave more precise inference of recombination rates than could be achieved from a single-generation cross, in particular in identifying recombination cold-spots. The recombination landscapes we describe have particular utility; both AILs are part of a resource to study complex yeast traits (see e.g. Parts et al., Genome Res 2011). Our results will enable future applications of this resource to take better account of local linkage structure heterogeneities. Our method has general applicability to other crossing experiments, including a variety of experimental designs.

## Introduction

**Figure 1:**
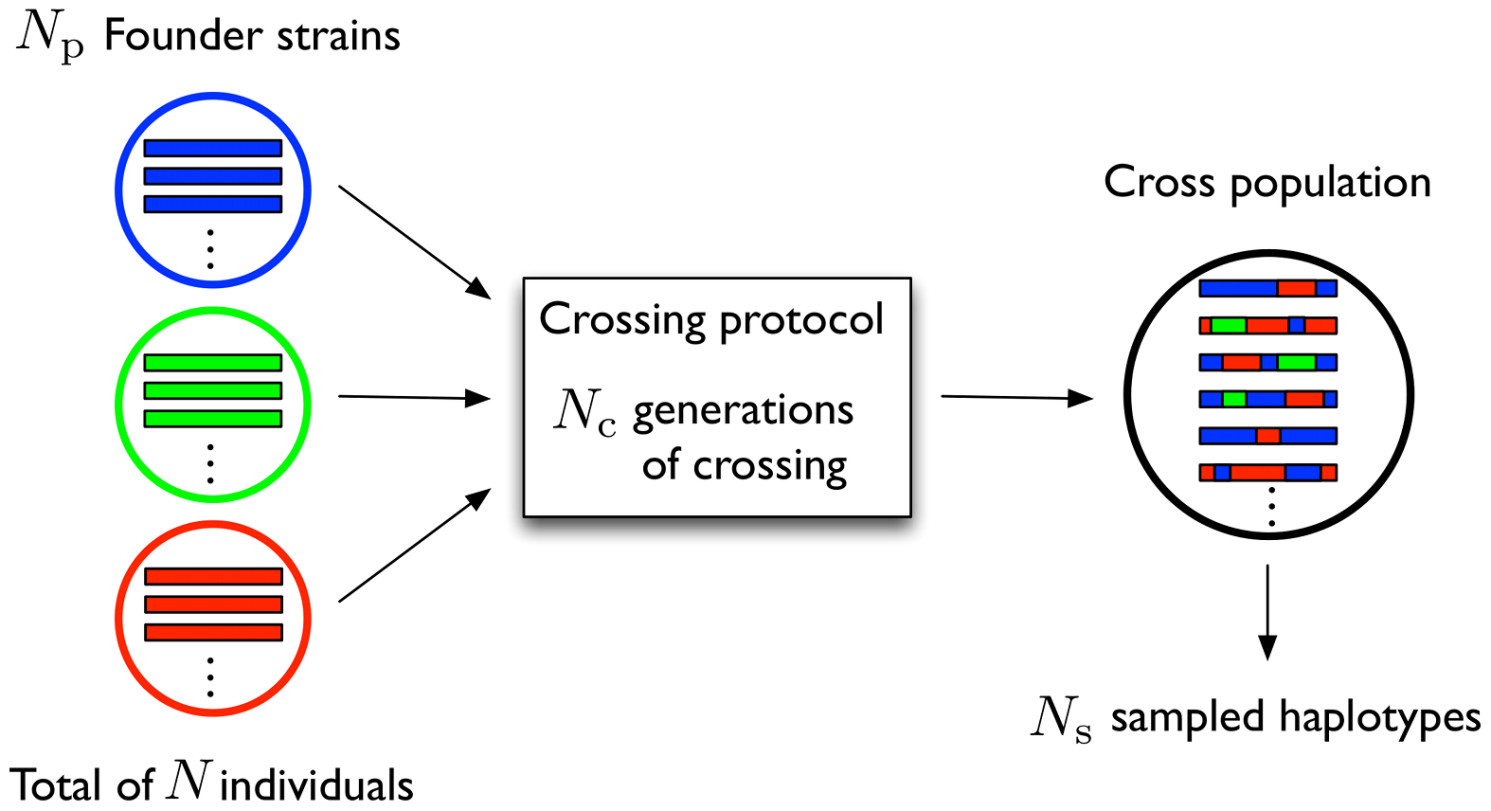
An example crossing experiment. The initial population has 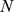 individuals drawn in some proportions from 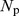 distinct parental strains. These individuals undergo 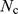 generations of random mating (multiple crossing protocols, including a funnel design, can be analysed), following which 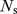 haplotypes are sampled. We use sequences from the offspring population to infer a recombination rate profile for the cross.

Accurate estimates of bare rates of evolutionary processes such as mutation and recombination are important building blocks in our understanding of evolution. These rates are known to vary across genomes; in the case of recombination, changes of orders of magnitude can occur between nearby loci at recombination hotspots [1]. As reviewed in depth elsewhere [2], [3], a range of methods have been employed to derive recombination rates from genome sequencing, including the use of pedigree information [4], [5], sperm typing [6], [7], and the application of methods from coalescent theory [8] to haplotype data [9]–[11]. Under this latter approach, probabilities are calculated of observing specific haplotypes under some mutation and recombination rate, these rates subsequently being estimated using, for example, maximum likelihood methods. This, however, only gives estimates of the bare recombination rates scaled by the effective population size, which is generally unknown. An alternative approach to learning recombination landscapes, which can lead to estimates of unscaled recombination rates, is that of genetic crosses of model organisms carried out at a large scale [12]–[16]. Where only two strains are involved in a cross, measurement of crossing over rates from individual sequences is straightforward, though where multiple strains are involved, such a calculation is more difficult. We here describe a new technique for inferring recombination rates from the sequences of offspring produced by a genetic cross with arbitrary initial strains, combining elements of the maximum likelihood techniques described above with known facts about the history of the cross population. We then apply our method to new whole genome sequence data from two yeast crosses. A typical experimental design, known as an advanced intercross [17], that produces data suitable for analysis using our method, is shown in Figure 1.

We consider an experiment in which a population of 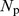 known parental strains, comprising 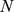 randomly mating individuals (other designs can also be incorporated), have undergone 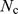 generations of crossing. We suppose that, from this population, the sequences of 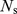 haplotypes have been sampled. Our calculation evaluates as a function of recombination rate the probabilities of observing, within the sample, specific two-locus haplotype frequencies. These two-locus haplotype probabilities are used to estimate the probabilities of observing 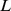-locus haplotypes, conditional on recombination rates, via a composite likelihood method [18], [19]. This likelihood function, together with the sampled sequences, can be used to infer local recombination rates.

We applied our method to two different examples of advanced intercross lines. The first example, described in detail in Ref. [20], consisted of twelve generations of random mating between West African (DBVPG6044, denoted WA) and North American (YPS128, denoted NA) yeast *Saccharomyces cerevisiae* strains. This was denoted the “two-way” cross, or by the sub-index 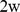. We here sequenced 2
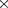
96 isolates from two biological replicates of the experiment to study the recombination landscape underlying the cross. The second example, which will be described in detail in a future publication (Cubillos et al. in preparation), consisted of twelve generations of mating, but with the addition of two extra parental strains, Wine/European (DBVPG6765, denoted WE) and Sake (Y12, denoted SA), to the design. This was denoted the “four-way” cross, or by the sub-index 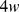. After a first generation in which only WA
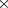
NA and WE
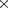
SA were allowed to mate the remaining eleven rounds were of random mating. Our analyses allowed a highly comprehensive view of general yeast recombination patterns and gave insights into specific differences between the crosses.

In a further step, we applied our method to data from the crossing experiment of Mancera et al. [14], from which genome-wide recombination in yeast was previously studied. In this cross, strains S288c and YJM789 were mated to give a diploid hybrid, subsequent to which all four spores from 51 meioses were genotyped at 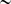
52 thousand markers (c.f. 52 thousand and 82 thousand segregating sites for the two- and four-way crosses). This design, denoted here as the “s-way” cross, or with sub-index 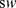, substantially differs from the other crosses. Firstly, whereas the two-way and four-way crosses had a large underlying population of 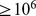 individuals, the s-way cross was derived from a single clonal hybrid. Secondly, the s-way cross was not produced via the same mating process, involving a single round, rather than multiple rounds, of mating. The non-random mating process in this case required a simple modification of our method; an assumption of random mating led to a recombination rate that was inflated by a factor of two (all first generation matings are between different parental strains, compared to only half of matings in the random case). The simplicity of this correction is due to the s-way design being a one generation cross.

We start by describing results obtained from the application of our inference method to simulated crosses. We then report the results of our genome-wide analysis of the cross-specific recombination profiles, and of our comparisons between them, in detail.

## Results

### Inferring recombination rates from simulated data

**Figure 2:**
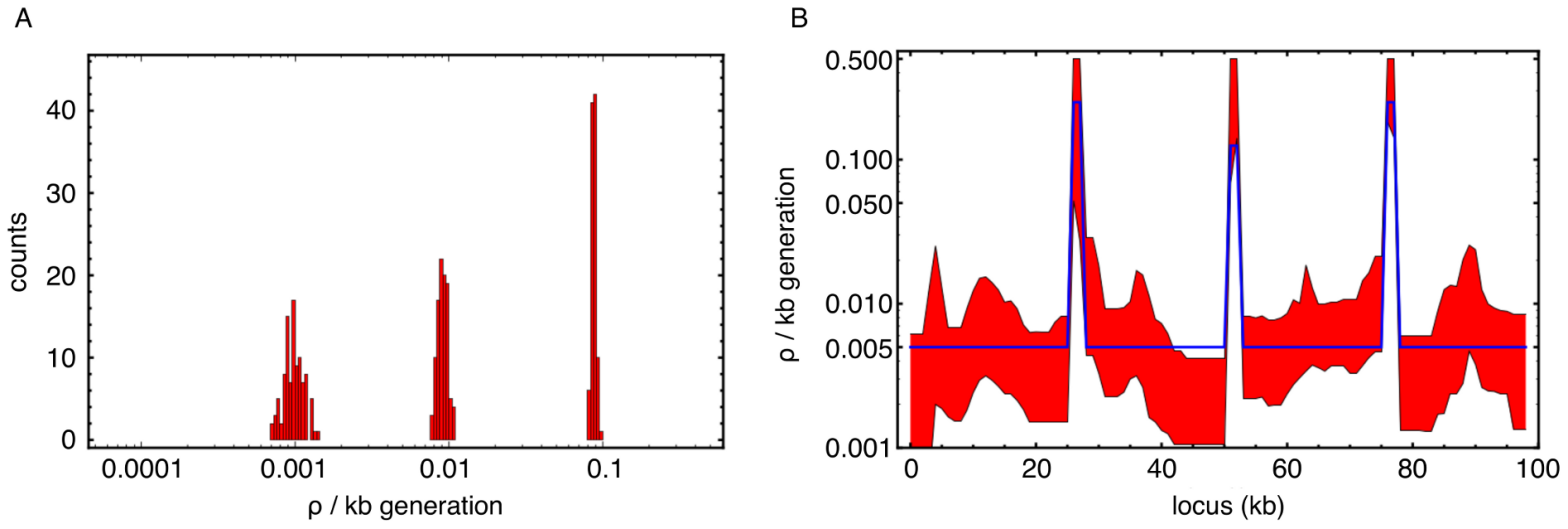
Inference of recombination from simulated data. a) A histogram of estimated recombination rates from simulated data under uniform recombination using our likelihood calculation together with the *interval* tool from *LDhat* [9]. Input recombination rates were chosen to cover a biologically realistic range 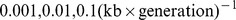 and were well recovered by the inference. Each of the 100 simulations has 100 segregating sites at 1 kb intervals (other parameters 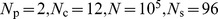). b) Inference of recombination rate for a simulation with varying rate. The simulated recombination profile (blue) had three recombination hotspots, with (50,25,50)-fold higher rate than the background; other parameters as before. The inferred profile is in good agreement with the input (red band: 95% confidence interval from 300 bootstrap samples of the single realisation of the crossing simulation).

Our method gave accurate inferences of recombination rates for simulated systems. Inferences of recombination were made for simulated data of an 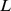-locus system (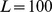) with uniform recombination rate (Figure 2a). The inference returned values close to the true value of the recombination rate 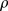, although with a slight underestimation, likely reflecting the composite likelihood approximation. Further inferences were made for a system including recombination hotspots (Figure 2b); again the true profile was closely replicated (empirical error estimates were derived via bootstrapping).

Our method was extended to cover finite populations by estimating the likelihoods of observing given haplotypes (see Methods, Eq. 3) via direct forward simulations. Comparisons of the likelihood surfaces for varying population sizes suggested that using the infinite population size limit is a good approximation for populations where 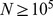 (see discussion in [21]). However, the infinite population size analysis produced comparable results to those of Figure 2a even in smaller populations, albeit with increased variance in the rate estimates (See Figure S1 in Text S1). This consistency in performance between small and large populations implies that, for the crossing parameters considered, the error induced by our assumption of infinite population size does not substantially outweigh the error stemming from the composite likelihood approach.

Our results from simulated data demonstrate the basic ability of the method to infer simple recombination profiles from offspring sequences of genetic crosses. Later in the text, we return to the issue of the consistency of results obtained with our model when applied to biological recombination landscapes.

### Genome-wide statistics of recombination

We first used our method to infer recombination rates for each of the two advanced yeast intercross lines. The mean recombination rate, measured genome-wide, was substantially higher in the four-way cross, at 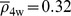 cM/kb, compared to 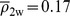 cM/kb for the two-way cross. The inferred value for the two-way cross provides an interesting comparison against a previous result, derived for the same system. On the basis of changes in allele frequencies resulting from exposure to heat stress (no haplotype data was available at that time), we inferred recombination rates for 44 regions of the genome identified as containing variants that conferred heat tolerance [22]. The mean of these rates was 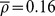 cM/kb, within 10% of the value reported here.

Analysis of data from the s-way cross of Mancera et al. [14] gave a mean recombination rate of 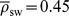 cM/kb, a factor of 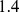 higher than that of the four-way cross. To make a direct comparison to what was previously reported for this population [14], we combined their previous genome-wide estimates for crossover and non-crossover rates (their Figure S5 in Text S1), and converted the results into our units (per haploid pair rather than per tetrad, and per kb rather than per base pair). At short distances, the effective recombination rate between two loci (measured by effect on linkage disequilibrium) is equal to the sum of the crossover and non-crossover rates, being dominated at large distances by the crossover rate. Applying a scaling factor between 0 and 1 to the reported non-crossover rate gave a range for 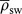 between 0.305 and 0.475 cM/kb; our estimate, calculated over a range of distance scales, lies towards the top of this range.

**Figure 3:**
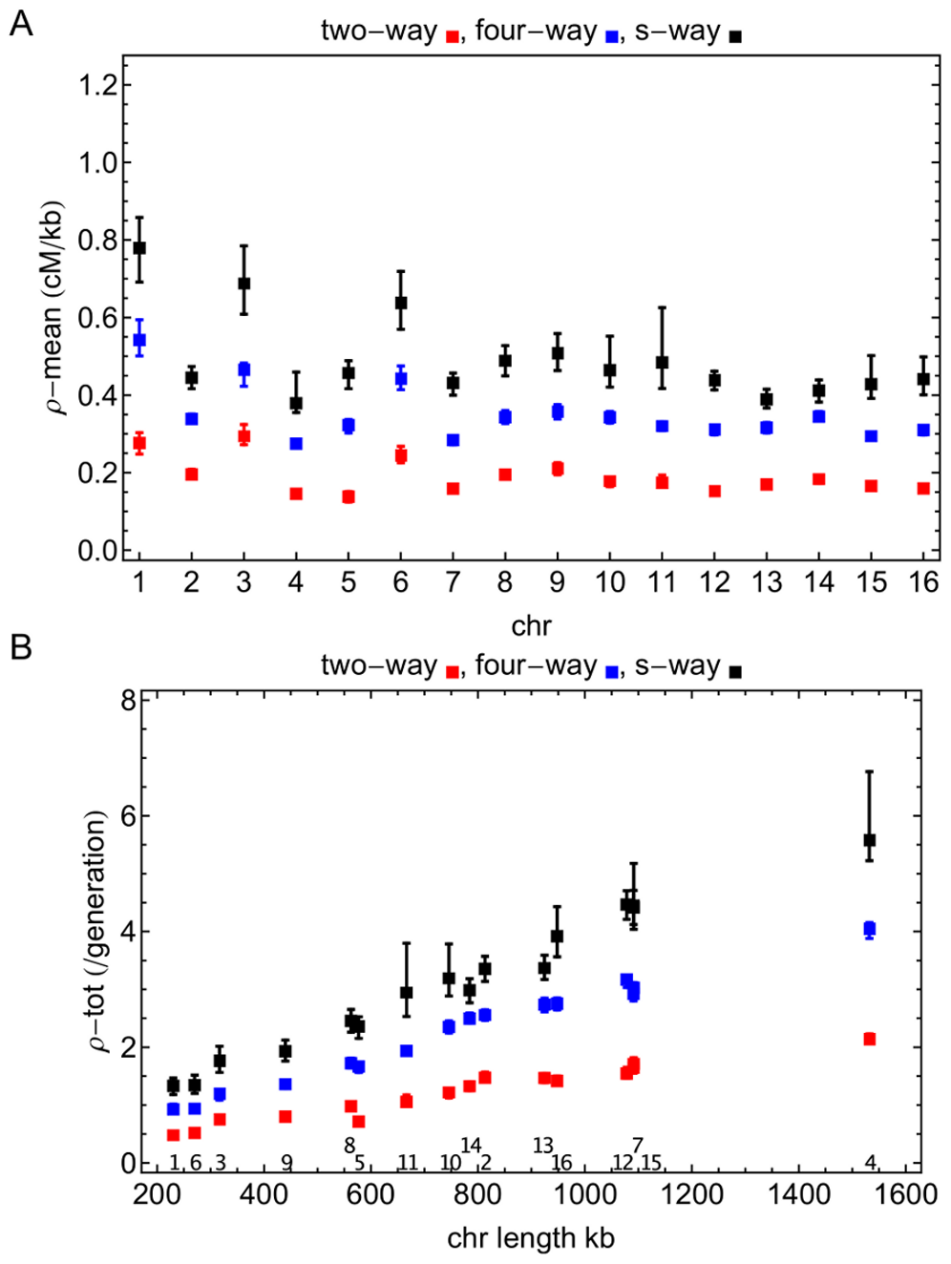
Recombination across yeast chromosomes. a) Mean (cM/kb) and b) total (per generation) rates of recombination across the chromosomes (chr number given above x-axis). There is substantial variability across chromosomes, the total rate of recombination correlating strongly with chromosomal length. Red, blue, and black squares denote values for the two-way, four-way, and s-way crosses respectively. Error bars show 95% confidence intervals evaluated via inferred recombination landscapes for at least 100 bootstrapped datasets. The multiple generations of recombination led to tighter estimates of recombination for the two-way and four-way experiments.

Investigating recombination across chromosomes revealed substantial variability in mean rates (Figure 3a) with the highest rates occurring in chromosomes 1, 3 and 6 for all crosses. Total rates of recombination per chromosome per generation are shown in Figure 3b). Total rates show a strong positive correlation with chromosomal lengths (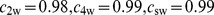), recapitulating known yeast biology [23]. The multiple generations of recombination included in the two-way and four-way crosses lead to more precise estimates of chromosome-wide rates of recombination, substantially lowering the variance in each estimate.

### High-resolution statistics of recombination

**Figure 4:**
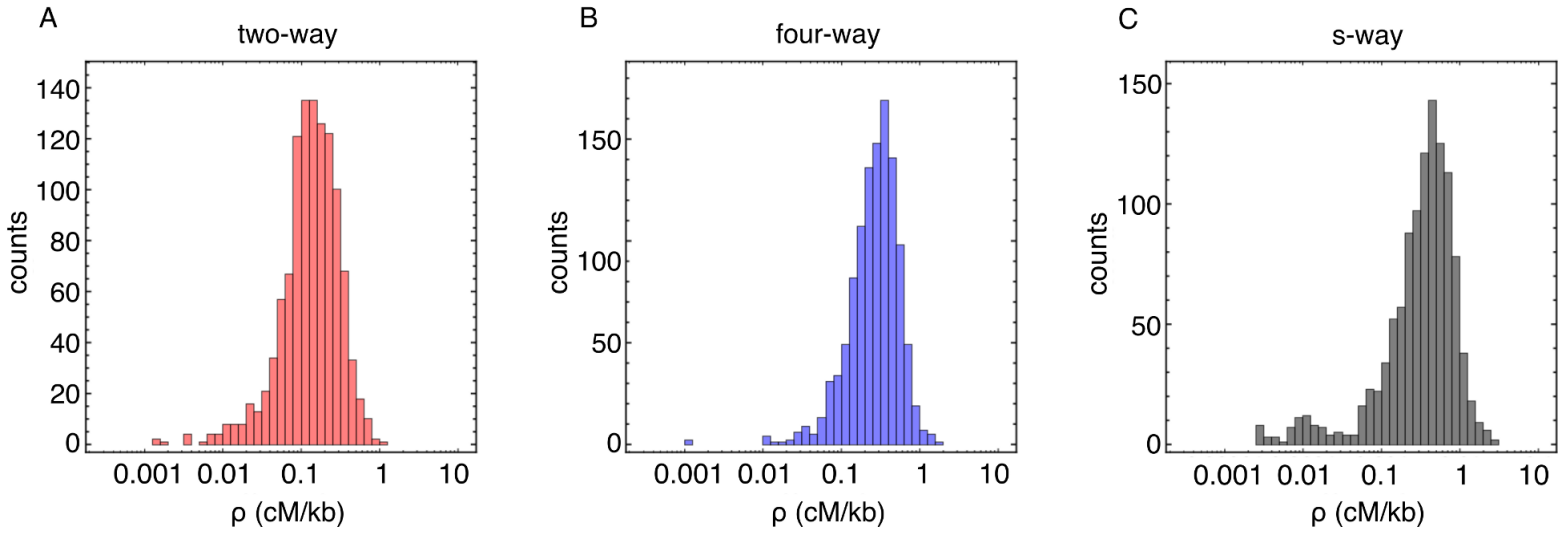
Genome-wide recombination rates at 10 kb resolution. Inferred recombination rates shown at 10.0 kb resolution across the genome from individuals generated through the two-way (panel a, red), four-way (panel b, blue) and s-way (panel c, black) crosses. The histograms show a broad distribution of recombination rates for each cross. The four-way cross had a substantially higher mean rate of recombination than the two-way cross, the s-way cross having a higher mean rate than the four-way cross (ratio of means 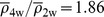 and 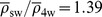).

Sequence polymorphisms between the parental strains gave more than fifty thousand segregating sites for the two-way cross, and more than eighty thousand for the four-way cross. Segregating sites were dispersed fairly uniformly across the genome (see Text S1 for more information on the data). Combining our likelihood calculation with optimisation routines from the widely used recombination rate estimation program *LDhat* [9], we obtained an estimated recombination rate for each interval between two consecutive segregating sites. These estimates were mapped onto uniform grids at varying scales 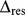 (0.5, 1.0, 2.0, 5.0 and 10.0 kb). Genome-wide, a broad spectrum of recombination rates was observed for genomic regions within all three crosses. Figure 4 shows recombination rates for these crosses measured at 10 kb resolution. For the two advanced intercrosses, our inferred distributions of recombination rates are unimodal and show a range of recombination rates, in this sense being similar to estimated distributions of recombination rates in humans [24]. Large differences between local recombination rates were evident genome-wide, with greater variance in the two-way cross; at 10.0 kb resolution a 95-fold difference was observed between 99% and 1% percentile rates inferred for the two-way cross, while a 38-fold difference was observed for the four-way cross. The s-way cross has what appears to be a bimodal distribution with a cluster of values around a recombination rate of 0.01 cM/kb; later we demonstrate that this is likely to be an artefact reflecting a lack of statistical power. As such, the very large 460-fold difference between 99% and 1% percentiles inferred for this cross is unlikely to reflect the true range.

#### Recovering known associations

Examining inferred recombination rates genome-wide, previously reported associations with regions of high recombination [25] were reproduced. For both the two-way and four-way crosses, the G+C nucleotide content of the 100 genomic regions (of length 5.0 kb) with highest recombination rate was significantly higher than the average G+C content for the whole genome (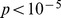, compared to random sets of 100 genomic regions). However, the effect size was very small, with an increase of only 2.1 percentage points in the average G+C content for the two-way cross, and 2.0 percentage points in the four-way, very close to the standard deviation in G+C content of individual 5.0 kb regions (2.1% in each case).

Further, a significant decrease in recombination rate was seen close to the centromeric regions of each chromosome [25]. In the two-way cross, the mean recombination rate at loci within 30 kb of the centromere was just over 60% of the mean rate across the genome (0.10 compared to 0.17 cM/kb, 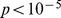). In the four-way cross, the mean recombination rate close to the centromere was close to 65% of the mean across the genome (0.21 compared to 0.32 cM/kb, 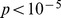). Further details are given in Text S1; see Figures S2 and S3 in Text S1.

#### Quantifying the shape of the landscapes

**Figure 5:**
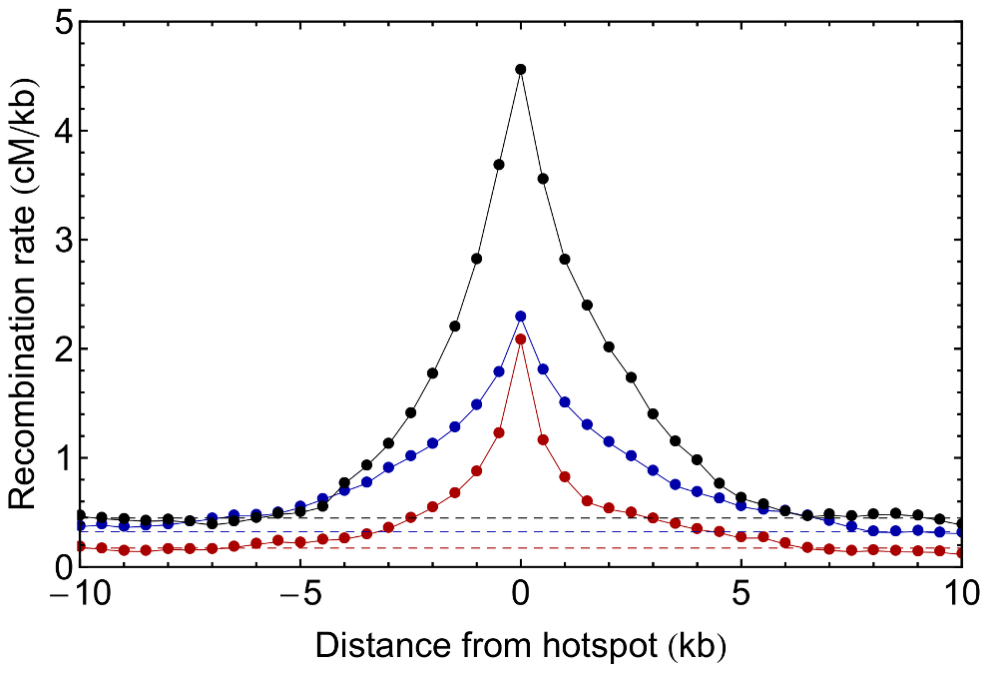
Decay of recombination rates near hotspots. Dots show the mean recombination rate for a 0.5 kb region at given distance from a region of high recombination rate in the 2-way (blue), four-way (red) and s-way (black) crosses. Statistics are calculated for genomic regions close to the 100 0.5 kb regions of highest inferred recombination rate. Dotted lines show the mean genome-wide recombination rate in each case (for congruence of hotspots see Figure S4 in Text S1).

Each of the crosses exhibited a highly rugged recombination landscape. The width of regions of raised recombination around hotspots was measured by calculating the mean recombination rate for 0.5 kb regions within set distances from each of the 100 such regions with the highest recombination rates. A return to genome-wide mean rates was observed at a distance of roughly 6.5 kb in the two-way cross, around 8 kb in the four-way cross, and about 6.5 kb in the s-way cross (Figure 5). These values were consistent with the overall statistics of autocorrelation within each recombination landscape. Autocorrelation functions were used to evaluate the overall variability of the landscape (measured at 0.5 kb resolution), fitting inferred rates to the functional form 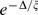, where 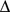 is the distance between sites and 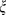 is the correlation length-scale in units of kb. Across the genome, these statistics had mean values 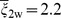 kb (std 1.0), 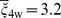 kb (std 0.9) and 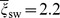 kb (std 0.5), indicating rapid changes in recombination rate between nearby sites in the genome.

#### Comparing the landscapes at different scales

**Figure 6:**
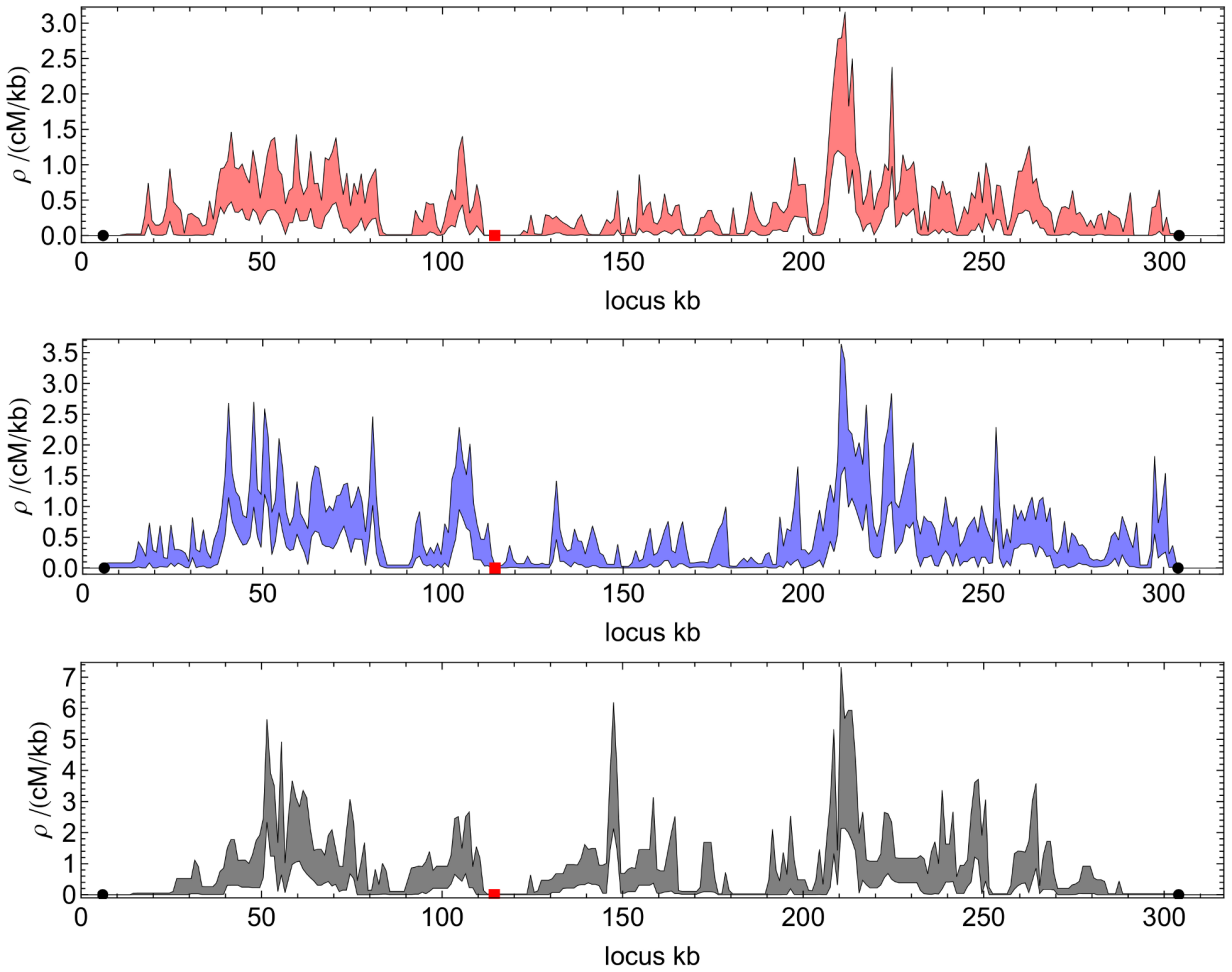
Recombination landscapes of chromosome 3 at 1 kb resolution. Recombination rates within chromosomes are highly variable. a) red: two-way b) blue: four-way cross, and c) black: s-way cross (90% confidence interval obtained by bootstrapping). We note the variable range of the vertical axis for each case. Red squares denote the centromere and black circles the first and last segregating sites for each cross; we cannot measure recombination between the chromosome ends and these sites.

Inferred recombination rates showed a relatively high level of congruence between crosses. Figure 6 depicts landscapes for the crosses across chromosome 3 at 1.0 kb resolution: the picture is similar for other chromosomes, with recombination across each chromosome varying strongly. The observed similarity between landscapes is consistent with reports of conservation of hotspots between divergent species of yeast [26]. However, considered in their entirety, the results inferred for the two- and four-way crosses were not fully consistent with their sharing a single underlying recombination landscape. Ten two-way crossing experiments were simulated, in which each population recombined according to the recombination landscape inferred for the two-way cross. Correlations between recombination landscapes inferred from each of these simulations were calculated, giving a measure of the expected congruence of landscapes inferred from a single underlying recombination profile. Although with increasing observation scale, 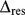, an increasingly strong correlation was seen between the real two- and four-way landscapes, the differences between them were greater than would be expected to arise from statistical noise alone (Table 1). Deviation between the landscapes was largest at the lowest scales 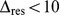 kb, with the 10 kb value almost overlapping with our expectation from simulations.

**Table 1:** Inferred landscapes are fairly congruent.

Correlation					
	Resolution (kb)				
	0.5	1.0	2.0	5.0	10.0
2-way cross vs. 4-way cross	0.55	0.61	0.68	0.77	0.81
Expected range (12-gen vs. 12-gen cross)	[0.72, 0.78]	[0.74, 0.8]	[0.78, 0.84]	[0.82, 0.88]	[0.81, 0.9]
2-way cross vs. s-way cross	0.39	0.44	0.51	0.58	0.63
4-way cross vs. s-way cross	0.48	0.52	0.59	0.65	0.69
Expected range (12-gen vs. 2-gen cross)	[0.46, 0.53]	[0.51, 0.58]	[0.57, 0.64]	[0.64, 0.72]	[0.67, 0.78]

The two-way landscape shows some deviation from the others.

Simulations were used to calculate the expected variability between landscapes inferred from replicates of a single crossing experiment. The inferred landscape for the two-way cross was used as an input recombination profile. The expected correlation range between landscapes for the 2-way and 4-way crosses was inferred from 10 simulation experiments, each with 12 generations of crossing. The range between minimum and maximum correlations is shown. Correlations between the inferred landscapes are high, but systematically smaller than the range from simulations. The expected correlation range between landscapes for the 2/4-way and s-way crosses was inferred as above from 10 simulation experiments, comparing a two-generation cross (mimicking the s-way design) with a 12-generation cross. Correlation values between the two and s-way crosses fall below this range, though correlations between the four and s-way crosses are consistent with the expectation.

Congruence between the recombination landscapes of the two- and s-way crosses was also lower than the expectation. Here, the design of the s-way cross, of a single round of crossing with non-random mating, was mimicked by simulating a two-generation random cross. Similar to the comparison between two- and four-way crosses, a small but visible deviation from the expected range was observed. Differences in recombination landscapes have previously been observed in crosses between different strains within a single species [16]. Our results suggest the presence of real differences between the two-way and the other crosses in the underlying, rather than simply the inferred, recombination landscapes.

Comparison of the results from the four-way and s-way cross gave correlations that were consistent with a shared underlying recombination landscape. This result does not imply that the underlying landscapes are identical, merely that any differences, should they exist, are not large enough to be detected under this genome-wide analysis. All three landscapes are shown in circos format [27] in Figures S6, S7, S8, S9 in Text S1. The relatively low level of correlation at 0.5 kb resolution between biological (and simulated) experiments highlights the level of statistical challenge in estimating recombination rates at high genomic resolution.

### Study design and the veracity of inferred recombination rates

**Figure 7:**
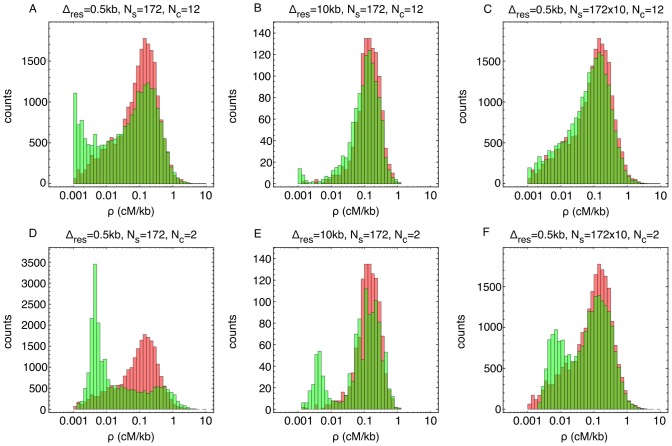
Assessing the robustness of inferred recombination characteristics. Red histograms show input data (two-way cross) and green inferred values from simulated crossing experiments using the two-way recombination landscape as input. The overlap between distributions is yellow-brown. a) At high resolution 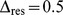 kb the inference is underpowered to call the low values leading to a systematic bias for these cold regions. b) Using 10 kb resolution removes this bias almost completely. c) The inference would also work for 0.5 kb resolution for the whole range of recombination rates if we would get ten times more samples. d–f) Analogous figures for a simulated two generation cross show that the bias is much larger and would persist even with ten times more data available.

Our ability to infer recombination rates was dependent upon the number of generations in the cross, the number of individuals sequenced, the resolution at which an inference was performed, and upon the underlying recombination rate in a given part of the genome. The inferred two-way cross recombination landscape was used as input for simulated crossing experiments encompassing twelve generations; we applied our inference method to see if we could infer it back. Trying to reproduce the histogram of Figure 4 was instructive and clearly showed that at resolutions 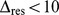 kb there is a systematic bias towards the lowest recombination rates (Figure 7a); for such resolutions the inference is underpowered. This systematic bias is almost fully removed at 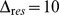 kb (Figure 7b); this was the rationale for using this scale in Figure 4. One remedy for getting accurate measurements at high resolution for the lower tail of the genome-wide recombination distribution would be to sequence 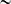
10 times more isolates. If we take the mean of the inferred recombination landscapes from the ten replicate crossing simulations, (given the large size of the underlying population more individuals could equivalently be sampled from a single experiment) we recover the correct distribution accurately for 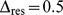 kb (Figure 7c).

By contrast, from crossing simulations that encompassed only one or two generations, we were not able to consistently reproduce the genome-wide recombination rate histogram, even at scale 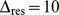 kb (Figure 7d–f). Using the two generation cross as a proxy for the s-way crossing design we conclude that the apparent bi-modality of the histogram inferred from the s-way cross (see Figure S5 in Text S1) most likely reflects a bias caused by lack of statistical power rather than any true biological signal. We note that these comparisons, at the level of histograms, are probing systematic inference errors over the whole range of recombination rates; consistency between the input vs. inferred distributions does not change the fact that error bars for any one interval are substantial, especially for the low recombination rates investigated at high genomic resolutions.

### Comparison with a high-resolution Double Strand Break point map

Trying to understand recombination is not only limited to observing recombination outcome. Meiotic recombination begins with the formation of double-stranded breakpoints (DSBs), which are resolved into recombination events. DSBs are themselves non-randomly distributed across the genome [28], [29]. Recent work has mapped their distribution across a yeast genome [30], allowing for comparison between sites of DSB occurrence and the subsets of those subsequently undergoing crossover or non-crossover events [31].

We took a DSB map [30], converted it to our scales 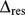, and compared it to our other landscapes. Conversion tracts in recombination events in yeast are frequently more than 1 kb in length [32], with crossovers possible at either end, such that comparison of landscapes at resolutions of 2 kb or less may not produce meaningful results. However, correlations at the 5 kb and 10 kb resolutions were fractionally lower than those identified between the inferred recombination landscapes. Against the 2-way, 4-way, and s-way crosses, the correlations were 0.57, 0.62, and 0.49 respectively at 5 kb resolution, and 0.61, 0.65, and 0.51 respectively at 10 kb resolution.

The DSB map is extremely reproducible and thus has little statistical noise associated with it [30], suggesting a genome-wide difference between the DSB map and recombination landscapes. However, the biological meaning of this is not clear. One caveat in comparing DSB locations and our landscapes lies in the potential difference between crosses of outbred strains and events that occur within the same strain. Any results from comparison of the two should be interpreted with the necessary caution. Further, while the three studied crosses have an impressive number of genetic markers, this resolution is still at least two orders of magnitude lower than the single nucleotide resolution of the DSB study [30].

### Discovering the hottest and coldest regions of recombination

**Figure 8:**
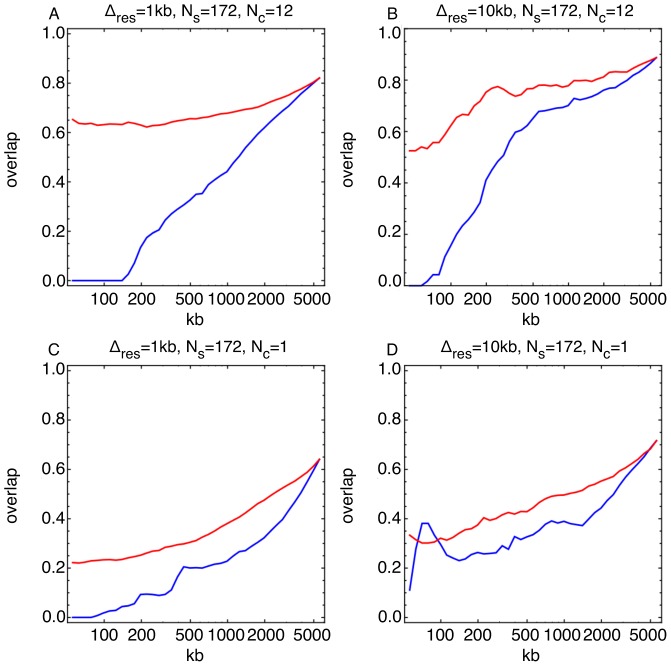
Power to discover recombination hot and cold regions under different crossing designs. Red (blue) curves show the ability to correctly recover the hottest (coldest) recombining 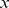 kb at resolution 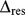 and number of crossing rounds 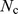. **a, b**) Results for an advanced intercross design, comprising 12 generations of crossing. Curves are calculated by comparing the locations of the hottest and coldest regions from the landscapes inferred for each of the ten simulated crossing experiments to the corresponding locations in the true input landscape (that inferred for the two-way cross), taking the mean value of the size of the overlap. **c, d**) Results for a single generation cross. The advanced intercross design has a clear advantage over single generation experiment.

Application of our inference method to the two advanced intercross datasets allowed us to investigate both the coldest and hottest regions of recombination with satisfactory true positive rates. Inferred landscapes from sets of ten simulated replicate experiments were used to measure the accuracy with which the hottest and coldest regions of recombination could be annotated. For each simulation, the two-way cross landscape was used as an input. Relative to a single generation cross, the advanced intercross design performed substantially better in identifying both the hottest and coldest regions (see Figure 8). From these results, we expected the protocol of the two and four-way yeast crosses to enable us to identify the coldest regions of the yeast genome with a decent statistical power. To test this, we took the coldest inferred 300 kb (at 1.0 kb resolution) from both of these crosses. These regions overlap by fraction 0.3 (i.e. 90 kb), moderately better than would be expected from our simulations.

## Discussion

Here we have described a bespoke method for recombination rate estimation in advanced intercross lines. Whereas a coalescent-based calculation for the haplotype likelihoods arises naturally in the context of wild populations with unknown histories, our “forward-in-time” analysis is intuitive for a crossing experiment with known initial population. Comparing our approach to that applied by Mancera et al. [14], we identify different advantages in each. Use of an advanced intercross design gives a more accurate picture of the fine structure of the recombination landscape, observation of a larger number of events translating into smaller error bars in the inferences. However, the Mancera et al. design has the substantial additional benefit of allowing the assignment of recombination events to non-crossover and crossover categories; something which cannot be achieved using our approach. Previously, in crossing experiments, custom-made tools have been developed to assign, at genome scale, every allele to a founder strain [15], [33] but to our knowledge these analyses have not so far focused on fine-scale recombination inference.

### Considerations for study designs

When studying recombination rates in the setting of a genetic cross, there are several experimental parameters and tradeoffs to consider. Key among these are the number of isolates (with associated cost in sequencing and isolate generation), the number of crossing rounds, typical inter-marker distances, the number of parental lines, and the population size. In order for the inference method to be able to faithfully capture the statistics of the whole range of recombination rates over the genome at the level of the histograms shown in Figure 4 we had to observe the rates at a scale of 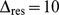 kb for the two-way (for the four-way cross with higher overall recombination rate we have better resolution). Estimates for each segment of the genome have uncertainties attached to them, which can be large for cold regions, but at this level we did not see a substantial systematic bias due to lack of statistical power. Based on simulations, having ten times more isolates would allow us to drop this scale to 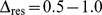 kb. Were the sequencing of thousands of isolates at reasonable cost to become a possibility, increasing the number of isolates would be a straightforward way to obtain more faithful inferences at higher resolutions. The best achievable resolution is ultimately limited by the distances between markers in the cross under study.

### Utility of fine-scale measurements of cross-specific recombination profiles

These two advanced intercrosses form part of a yeast resource to systematically study complex traits. We hope that the cross specific recombination landscapes that we inferred here will help future studies utilising this resource. For example, any quantitative trait loci (QTL) mapping study (for a review see [34]) will greatly benefit from a quantitative understanding of local recombination (and hence the linkage structure) of the specific cross. We have previously analysed such QTL selection data using a model where beneficial “driver” alleles for the selection condition were linked to neutral “passenger” alleles [22]. In that study, the movement of passenger alleles was dependent on the local linkage structure, which was modelled via a set of locally uniform recombination rates, learnt independently for different sections of the genome. Given the potential for recombination landscapes to be extremely rugged, incorporating fine resolution recombination rate maps will be critical to improve such inferences of QTLs. Finally, these landscapes allow for various data mining opportunities which have the potential to increase our biological understanding of the process of recombination.

### Possible reasons for the observed differences in recombination activities between the yeast crosses

Arguably the most striking difference between the inferred recombination profiles is the large difference in the overall rates: 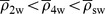. Given that the s-way design is very different to the advanced intercrosses we are perhaps less surprised that the overall s-way activity differs from the other two. For example, analyses of limited genotype data from chr 13 of the two-way cross collected after one, six and twelve rounds of crossing suggested that recombination may slow down as the cross progresses [30]. Our inference method does not consider the role of selection during the cross; if increased recombination led, in general, to individuals of lower fitness, then over repeated generations of crossing, a lower effective recombination rate would be observed. Further reasons may lie behind the difference between the two and four-way crosses, such as the different genetic distances between strains. In single-generation crosses involving these strains, differences between the numbers of recombination events were observed, the WA strain recombining less than the other strains [35]. Pinning down the effect of this upon the two crosses is difficult without also having measurements of the intra-strain rates. However, as a lower fraction of mating events in the four-way cross involve the WA strain (7/16 compared to 3/4), this would suggest an overall higher rate of recombination in the four-way cross.

### Future applications and extensions of the inference method

The inference framework we describe can be extended to more complex experimental procedures or populations (see e.g. [36]). While, for example, the protocol described in Figure 1 has an arbitrary number of parental strains, our calculations are straightforward to extend to protocols going beyond this, such as a funnel design [37] (See Text S1 for details). Our method would also be extendable to the estimation of recombination rates between different crossing rounds if isolate data were available for these intermediate states, allowing for the measurement of how the number of crossing generations affects recombination activity.

### Conclusions

We here developed a new approach for estimating fine-scale recombination rates from genome sequences of individuals sampled from genetic crosses. The method recovered accurately the correct profiles when applied to simulated crosses. Our application of the method to three specific yeast systems revealed interesting cross specific differences between the overall recombination activities. Our method is applicable to crossing scenarios other than those used for the studied yeast crosses. Finally, as the two and four-way crosses are an integral part of a genetic resource to study complex traits and genotype to phenotype maps in yeast, we hope that the fine-scale estimation of the recombination properties of these crosses will help to improve future studies using the resource.

## Methods

### Inference method

Given sequences of individuals resulting from a crossing experiment, the recombination rate between any two loci can be inferred via a simple likelihood calculation. We begin by assuming a two-allele-per-locus model; this assumption generally holds due to the low per-locus mutation rates in biological systems (in practise, loci with more than two alleles can be omitted without losing any substantial amount of data).

Considering loci 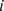 and 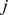, each with two alleles (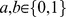), in a population of infinite size, the haplotype frequencies, 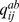, after 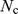 generations of random mating, can be expressed analytically as
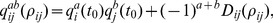
(1)where, for example, 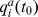 is the initial frequency of allele 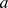 at locus 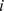, and 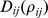, the linkage disequilibrium between alleles at loci 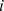 and 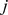 after the cross, is given by [8]:
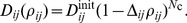
(2)Here 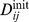, the initial linkage disequilibrium between 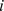 and 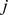, depends on the initial haplotype frequencies 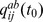, 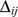 is the distance between the loci 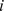 and 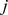 (in units of kb), and 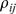 measures recombination between the loci in units of 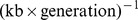. We use 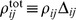 to represent the total rate of breaking of linkage between 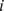 and 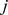; separation of rates of crossover and non-crossover events is not considered in this paper.

We note that, in the above equations, all except two of the parameters are known from the locus positions and the structure of the cross, the exceptions being the recombination rate, 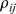, and the set of final haplotype frequencies, 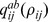 (knowledge of one these specifies a value for the other). Sequencing 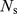 isolates from the offspring population gives the observed pair-wise haplotype counts 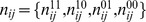. From these, an expression can be written for the likelihood of a given set of underlying values of 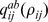, and hence for the likelihood of a given recombination rate between 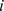 and 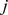.
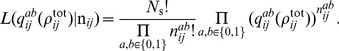
(3)In this manner, we can derive a maximum likelihood estimate of the recombination rate between 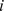 and 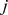. We note that, in the case of a finite population, Eqs. 1 and 2 are no longer exact, representing instead the expected outcome of recombination. In the finite population size case, the likelihood of Eq. 3 can be estimated by means of direct forward simulations. Recombination rates are estimated by our code in units of 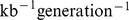, but are reported above in units of cM/kb.

Estimation of recombination rates across regions spanning multiple loci was conducted using a composite likelihood approach. For multi-locus systems, deriving analytical probability functions equivalent to Eq. 3 remains an outstanding challenge for population genetic theory, while numerical approaches for sampling the appropriate full likelihood surfaces are currently impracticable for genome scale analyses. For these reasons, the full likelihood function is often evaluated in an approximate way using the composite likelihood approach (that is, calculating the product of all pairwise probabilities. Often only a limited number of consecutive sites (e.g. 50 in *LDhat*) are used in the computation to improve the speed of calculation) [18], [19]. Results obtained from composite likelihood methods are often good, though the approximate nature of the probability complicates, for example, the estimation of confidence intervals for the inferred model parameters [1], [38]. Following this method, we combined our likelihood calculation with an optimisation routine from the existing recombination rate estimation software *LDhat* [9].

### Data sets

Details on isolation and sequencing of individual segregants and variant calling can be found in Text S1.

### Data access

The inference program, haplotype data, and inferred yeast landscape are available from ftp://ftp.sanger.ac.uk/pub/teams/153/AIL-yeast/. Sequence data is available from European Nucleotide Archive (ENA) under access number ERP000780.

## Supporting Information

Text S1 **Details of isolation and sequencing of individual segregants, calling of segregating sites, and genotyping from sequencing data.** Discussion of the potential to incorporate other breeding designs to the inference method. Details of crossing simulations, including results from use of the infinite population size approximation with small populations. Details of the recovery of known associations with recombination rate. Decay or recombination rate close to hotspots, and congruence between the crosses. Genome-wide reproductions of landscapes inferred for each of the crosses. Figures S1 to S9 are contained within Text S1.
(PDF)
