# Supplementary material for: Inferring Genome-Wide Recombination Landscapes from Advanced Intercross Lines: Application to Yeast Crosses
Source: PLoS One. 2013 May 2;8(5):e62266. doi: 10.1371/journal.pone.0062266 (PMC3642125; doi:10.1371/journal.pone.0062266)
Supplement: Text S1 — Details of isolation and sequencing of individual segregants, calling of segregating sites, and genotyping from sequencing data. Discussion of the potential to incorporate other breeding designs to the inference method. Details of crossing simulations, including results from use of the infinite population size approximation with small populations. Details of the recovery of known associations with recombination rate. Decay or recombination rate close to hotspots, and congruence between the crosses. Genome-wide reproductions of landscapes inferred for each of the crosses. Figures S1 to S9 are contained within Text S1. (PDF) [file pone.0062266.s001.pdf]

Supporting Text: Inferring genome-wide recombination landscapes from advanced intercross lines: application to yeast crosses

1 Data generation and preprocessing

1.1 Isolation and sequencing of individual segregants

We sequenced 192 segregants isolated from an advanced intercross line (F12) between *S. cerevisiae* strains YPS128 and DBVPG6044 [1]. The F12 diploid pools, from replica 1 and 2, were sporulated in KAc for 10 days. Approximately 90% of the cells produced meiotic segregants (spores) contained in ascus. Non sporulated cells were killed by ether treatment and the ascus was digested for 1 hour using 100 μl of Zymolase (10 mg/ml) [1]. Cells were washed in sterile water and vortexed vigorously for five minutes to disperse the spores. Serial dilutions were plated in YPDA to isolate single colonies. Segregants were genotyped for the mating type (using haploid tester strains), LYS and URA (using lys and ura drop out media respectively). For each replica (1 and 2), we selected 48 Mat α, URA + segregants (genotype Mat α, ho::HygMX4, ura3::KanMX4, lys2::URA3) and 48 Mat a, LYS + (Mat α, ho::HygMX4, ura3::KanMX4). We extracted DNA from the 192 segregants using the MasterPureTM DNA Purification Kit (Epicentre) using a slightly modified protocol from the one indicated by the manufacturer. Cells were inoculated in 1 ml liquid YPD at 30°C in 1.5 ml Eppendorf tubes without shaking. The overnight cultures were washed in distilled water and resuspended in 200 μl of 50 mM EDTA and 20 μl of Zymolase (10 mg/ml) were added. Cells were incubated at 37°C per 30 minutes to obtain spheroplast. The manufacturer protocol was followed thereafter. 50 μl of TE buffer were added to the DNA pellet and incubated 20 minutes at 65°C to facilitate resuspension. The 192 segregants for the 4-way cross were obtained in a similar way except that meiotic spores were isolated by micromanipulation (Cubillos et al., manuscript in preparation). The extracted DNA samples were sequenced using 2 lanes of Illumina HiSeq 2000 paired end sequencing each lane containing 96 multiplexed samples. The sequencing reads and the associated statistics are available in European Nucleotide Archive (ENA) under access number ERP000780.

1.2 Calling of segregating sites and genotyping from Next Generation Sequencing data

Reads for the parental strains YPS128, DBVPG6044, Y12 and DBVPG6765 were cleaned from adapter contaminants using cutadapt 0.9 (http://code.google.com/p/cutadapt/) and mapped to the *S. cerevisiae* S288C reference genome (Release R64-1-1) using Stampy 1.0.18 [2] in hybrid mode with BWA 0.5.9 [3]. Non-primary alignments and non-properly paired reads were filtered out and duplicate reads were removed using Picard 1.69 (http://picard.sourceforge.net/). Local realignment was performed on the four strains simultaneously using SRMA [4]. Base qualities were capped by their Base Alignment Qualities [5] as computed by SAMtools 0.1.18 [6]. Single-nucleotide polymorphisms were then called using FreeBayes 0.9.5 (http://bioinformatics.bc.edu/marthlab/FreeBayes) set for haploid samples. A list of segregating sites for the YPS128-DBVPG6044 cross was derived from the resulting list of SNPs by selecting sites that were covered by at least 10 reads in both strains and at most a number of reads equal to twice the genome-wide median coverage of each strain respectively, that had at most one read conflicting the consensus allele call and where the called genotypes were polymorphic between the strains. Similarly, a list of segregating sites for the cross involving the four parents was derived by selecting sites that passed these same filters applied to all four strains.

Reads for segregant strains were mapped to the S288C reference genome using Stampy and filtered for non-primary alignments, non-properly paired and duplicate reads as above. Strains showing signs

of diploidy or DNA contamination as assessed by genome-wide patterns of heterozygosity were excluded from further analysis. The strains were genotyped at the corresponding segregating sites using SAMtools mpileup 0.1.18, calling the respective genotype if the log ratio between the likelihood of the data as computed given homozygosity for the reference allele and the likelihood given homozygosity for the alternative allele was larger than 10 or smaller than -10, assigning missing data if in-between. As a further filter against heterozygosity resulting from mis-mapped reads and collapsed repeats, sites on which three or more segregants had a genotype likelihood ratio in-between 5 and -5 were removed from further analysis. This pipeline was applied to segregants from both the two-way and the four-way cross. After these filtering steps the two-way cross had 172 individuals and 52466 segregating sites and the four-way cross 175 individuals with 82910 segregating sites.

Additional filtering

Genuine subtelomeric translocation events (to be described elsewhere) led to false calls of high recombination at the ends of chromosomes 2, 7, 15, and 16. Furthermore, the relative variability of markers in the subtelomeric regions between the crosses would complicate the correlation analysis at these locations. For these reasons all recombination rates within 30kb of the ends of all chromosomes were cut off from the statistical analysis (although the whole ranges are plotted in the circos plots and given in the data). We note that the amount of recombination between the chromosome start and the first marker and between the chromosome end and the last marker cannot be inferred.

2 Incorporating other breeding designs to the inference method

The four-way cross was constructed such that during the first round only matings between WA×NA and WE×SA were allowed. This is similar to what is called the funnel design [7], albeit a very simple one at that. We outline here how our calculations can be augmented to factor in such experimental choices. Adapting the notation from Methods section to carry information of the specific branch of the cross we denote two locus haplotype frequencies after one generation of crossing as $q^{ab}_{ij,\mathrm{WA}\times\mathrm{NA}}(\rho_{ij})$ and $q^{ab}_{ij,\mathrm{WE}\times\mathrm{SA}}(\rho_{ij})$, where as before $q_{ij,\mathrm{WA}\times\mathrm{NA}}$ and $q_{ij,\mathrm{WE}\times\mathrm{SA}}$ are functions of the respective initial linkages, allele frequencies and recombination rate (assumed to be shared). Again, fixing values for ρ_{ij} fully fixes the haplotype frequencies (infinite population approximation), as other degrees of freedom are fixed by the cross at hand.

Before further rounds of crossing these haplotype frequencies will be mixed by some factor α which in this cross is 0.5:

$$q^{ab}_{ij,\mathrm{F1}}(\rho_{ij}) = (1-\alpha)q^{ab}_{ij,\mathrm{WA}\times\mathrm{NA}}(\rho_{ij}) + \alpha q^{ab}_{ij,\mathrm{WE}\times\mathrm{SA}}(\rho_{ij}) \quad (1)$$

From these numbers we can calculate the allele frequencies $q^{a}_{i,\mathrm{F1}}(\rho_{ij}) = \sum_{b\in 0,1} q^{ab}_{ij,\mathrm{F1}}(\rho_{ij})$ and linkage $D^{\mathrm{init}}_{ij,\mathrm{F1}} = q^{11}_{ij,\mathrm{F1}}(\rho_{ij}) - q^{1}_{i,\mathrm{F1}}q^{1}_{j,\mathrm{F1}}$ at this time point of the cross, these values depending (not written here explicitly) on the initial allele frequencies at both branches of the cross, the initial linkages at both branches of the cross and finally the recombination rate. But for the recombination rate, all of these are known. From this point, all that remains is to use the equations as given in Methods section for the remaining $N_{\mathrm{c}}-1$ generations, this time treating what was evaluated above as the initial condition.

More complex crossing designs can be incorporated in a similar manner, with the infinite population size approximation keeping the calculation simple. We note that for the particular four-way cross studied here, explicit modelling of the first round in the four-way cross did not affect the results in an appreciable manner. As such, all calculations were done using the fully random mating version of the code, which allows for an arbitrary number of parental strains and mixings.

3 Crossing simulations

We took all segregating sites from the two-way cross for each chromosome together with the inferred recombination rates to simulate the crossing protocol. We then created a population of N individuals where at the beginning half of the individuals were carrying alleles 0 at all segregating sites and the other half alleles 1. The population was put through N_c generations of random mating under the input recombination profile. For every generation, we drew randomly N/2 pairs of haploid parent genomes from the previous generation with replacement. For each such pair we drew a number of recombination events using a Poisson distribution with the total chromosomal rate to be sprinkled according to the appropriate spatial distribution (the input profile) across the chromosome. The resulting two haploid recombinants formed two new individuals for the next generation.

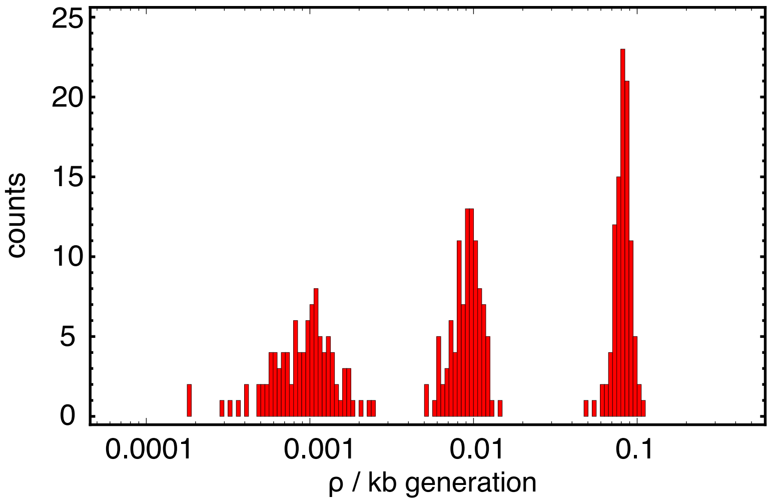

Figure S1. Using the infinite population size calculation to infer recombination rates from small populations.
A histogram of estimated recombination rates from simulated data under uniform recombination. Input recombination rates were chosen to cover a biologically realistic range $0.001, 0.01, 0.1(\mathrm{kb} \times \mathrm{generation})^{-1}$ and are well recovered by the inference. Each of the 100 simulations has 100 segregating sites at 1kb intervals (other parameters $N_\mathrm{p} = 2, N_\mathrm{c} = 12, N = 10^2, N_\mathrm{s} = 96$). The infinite population size calculation is still producing good results for the small population, $N = 10^2$, even if the overall variance has visibly increased when compared to the Main Text Figure 2a with $N = 10^5$.

4 Recovering known associations

4.1 High G+C content of hotspots

The 100 regions of highest recombination rate (at 5kb resolution, giving 500kb of sequence in total) were identified from the recombination map inferred for the 2-way cross. The mean G+C nucleotide content was then measured across these regions as 40.4%, higher than the equivalent value of 38.2%, measured across the genome as a whole. To test the significance of this result, 10^5 random sets of 100 regions were drawn from across the genome, and the mean G+C content measured for each. The G+C content of the

high-recombination regions was higher than that of any of the 10^5 random regions (Figure S3). Results for the 4-way data were extremely close to this with a GC content in the regions of highest recombination rate of 40.3%. For each calculation, the GC content of a region of the genome was calculated as the mean GC content for that region across all of the strains involved in the relevant cross; the values reported above are identical to those which would be calculated (to the given precision) without accounting for differences between strains.

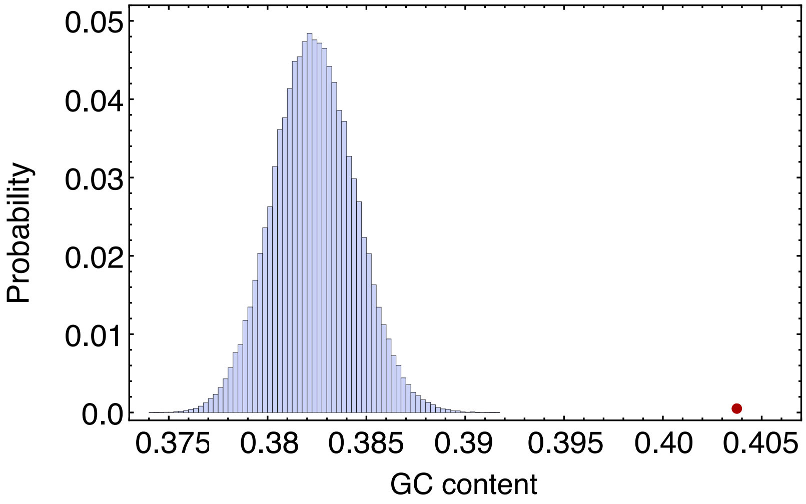

Figure S2. High GC-content associated with high recombination rates.
Histogram shows the distribution of GC content for 10^5 randomly selected sets of 100 5.0 kb regions of the genome. The mean GC content for the 100 5.0 kb regions of the genome with the highest recombination rates is shown as a red dot.

4.2 Low recombination rate near centromeres

Regions of the genome, measured at 0.5kb resolution, were classified according to their distance from the centromere, calculating the distance from the central point of each region to the nearest point of the centromere. For the two-way cross, the mean recombination rate for the 1922 regions 30kb or less from the centromere was 60.5% of the mean recombination rate across the genome; for the 4-way cross, the recombination rate was 64.5% of the mean genome-wide rate. The significance of each result was tested by drawing 10^5 random sets of regions from across the genome, and calculating the mean recombination rate across each; in this manner each result was found to be significant at $p \leq 10^{-5}$. Visual examination of recombination rates shows substantially lower rates near the centromere. An equivalent calculation for the s-way cross showed a mean recombination rate close to the centromere that was 72.4% of the mean genome-wide rate.

4.3 Decay of recombination close to hotspots

Recombination rates close to hotspots returned to genome-wide mean rates within a distance of roughly 6-8kb. The hottest regions of recombination identified in each cross were associated with increased rates of recombination in the other crosses. For example, in the 100 hottest 1kb regions identified from the 2-way cross, the mean recombination rates for the 4-way and s-way crosses were 1.13 cM/kb and 1.70

cM/kb respectively, 3.5 and 3.8 times higher than the mean rates for these crosses. In the 100 hottest 1kb regions identified in the 4-way cross, the recombination rates for the 2-way and s-way crosses were 2.11 cM/kb and 0.87 cM/kb, 5.0 and 4.7 times their respective mean recombination rates (Figure S4).

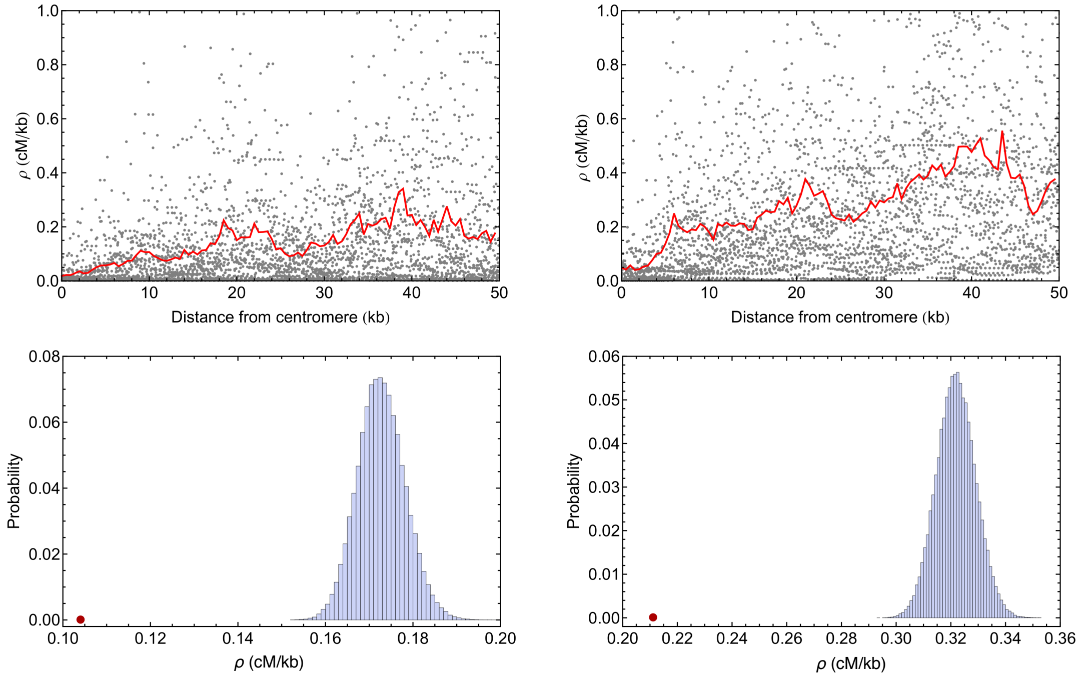

Figure S3. Low recombination rates close to centromeres.
Grey dots show recombination rates inferred for 0.5 kb regions for the two-way (top left) and four-way (top right) crosses. The red line in each case shows the mean recombination rate over windows of 0.5 kb from the centromere. Recombination rates for random selections of 1922 genome regions, each of 0.5 kb, are shown as histograms for the 2-way (bottom left) and 4-way (bottom right) datasets; the red dot in each case represents the mean recombination rate identified for the genome regions within 30 kb of the centromere.

References

1. Parts L, Cubillos FA, Warringer J, Jain K, Salinas F, et al. (2011) Revealing the genetic structure of a trait by sequencing a population under selection. Genome Res 21: 1131–8.

2. Lunter G, Goodson M (2011) Stampy: a statistical algorithm for sensitive and fast mapping of illumina sequence reads. Genome Res 21: 936–9.

3. Li H, Durbin R (2009) Fast and accurate short read alignment with Burrows-Wheeler transform. Bioinformatics 25: 1754–60.

4. Homer N, Nelson SF (2010) Improved variant discovery through local re-alignment of short-read next-generation sequencing data using srma. Genome Biology 11: R99.

5. Li H (2011) Improving snp discovery by base alignment quality. Bioinformatics 27: 1157–8.

6. Li H, Handsaker B, Wysoker A, Fennell T, Ruan J, et al. (2009) The sequence alignment/map format and samtools. Bioinformatics 25: 2078–9.

7. Churchill GA, Airey DC, Allayee H, Angel JM, Attie AD, et al. (2004) The collaborative cross, a community resource for the genetic analysis of complex traits. Nature Genet 36: 1133–7.

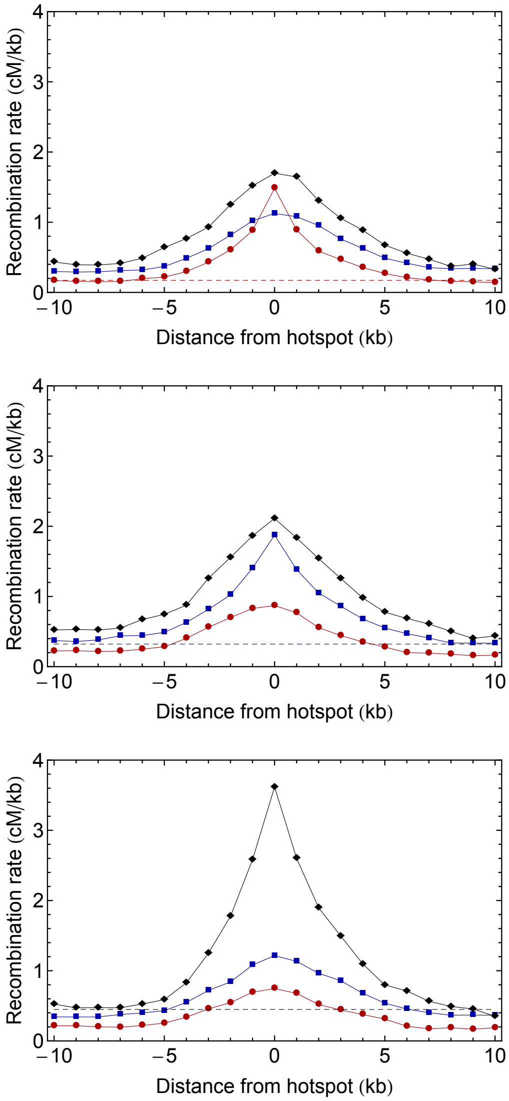

Figure S4. Decay of recombination close to hotspots and congruence between crosses. The 100 1kb regions with the highest inferred recombination rates were identified in the 2-way (top), 4-way (middle) and s-way (bottom) crosses. The mean recombination rate was calculated for each of these regions, and for 1kb regions up to a distance of 10kb in the genome from the hotspots. Each figure shows the mean recombination rates inferred for the cross, as well as mean recombination rates inferred for the same genomic regions in the other two crosses. Data is shown for the 2-way (red), 4-way (blue) and s-way (black) crosses in each case. Dotted lines show mean recombination rates for each cross respectively.

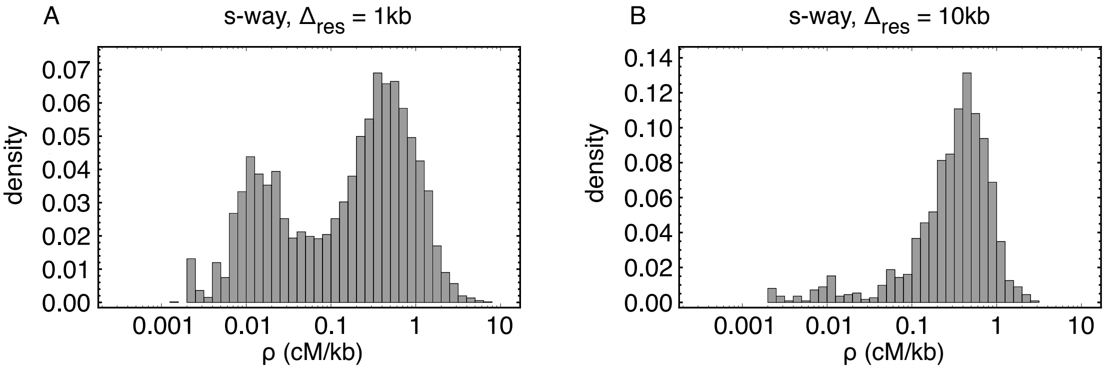

Figure S5. Genome-wide recombination rates for the s-way cross.
a) The distribution of recombination rates inferred for 1.0kb regions of the genome from individuals generated through the s-way cross has a second peak at low recombination rates. b) At 10kb resolution the second peak almost disappears. As discussed in the main text we believe the secondary peak at the low rates reflect the lack of statistical power in this range rather than representing a true biological signal.

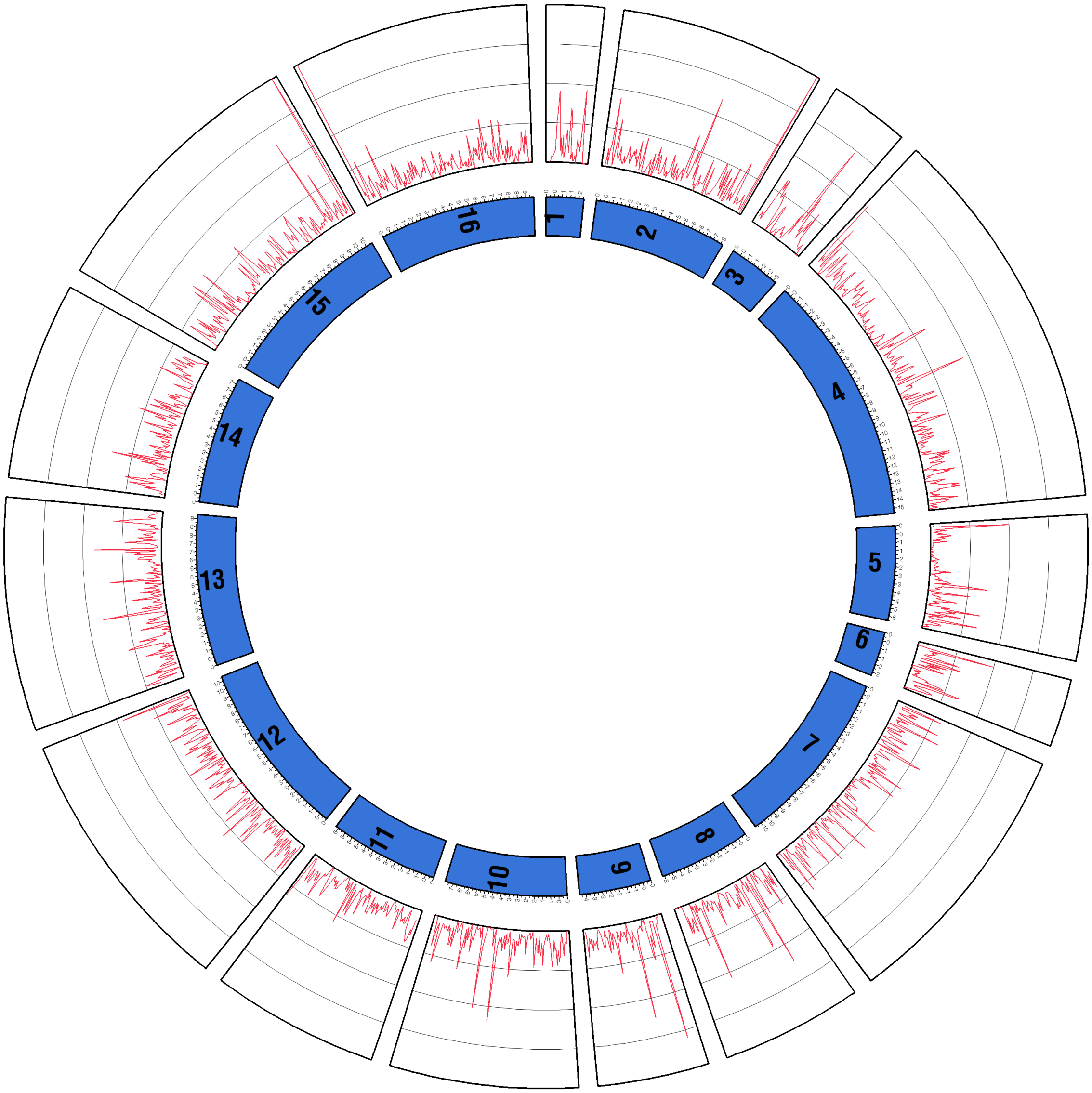

Figure S6. Recombination landscape of the two-way cross at 5kb resolution. The range for the y-axis is from 0 to 2 cM/kb.

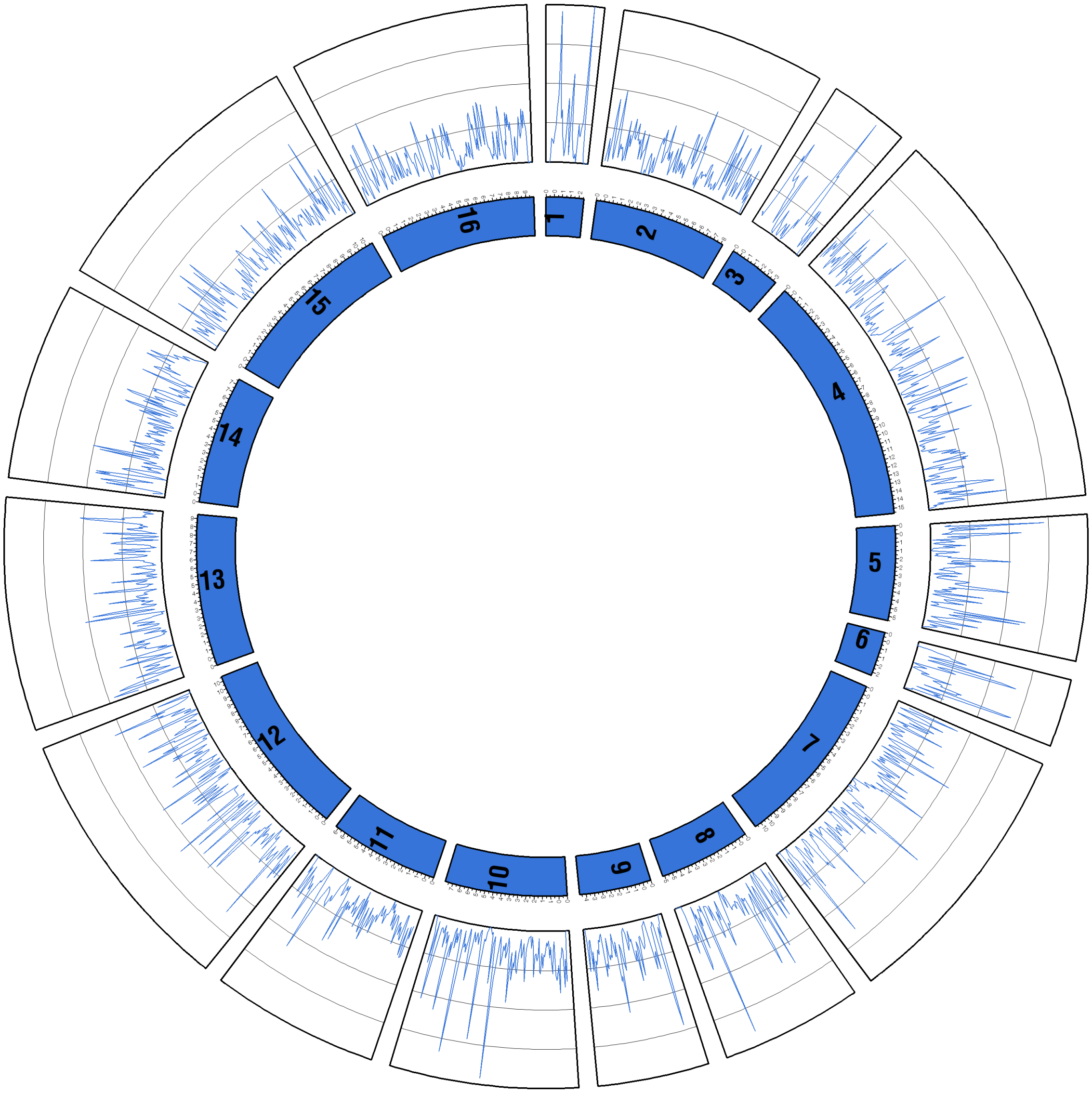

Figure S7. Recombination landscape of the four-way cross at 5kb resolution. The range for the y-axis is from 0 to 2 cM/kb.

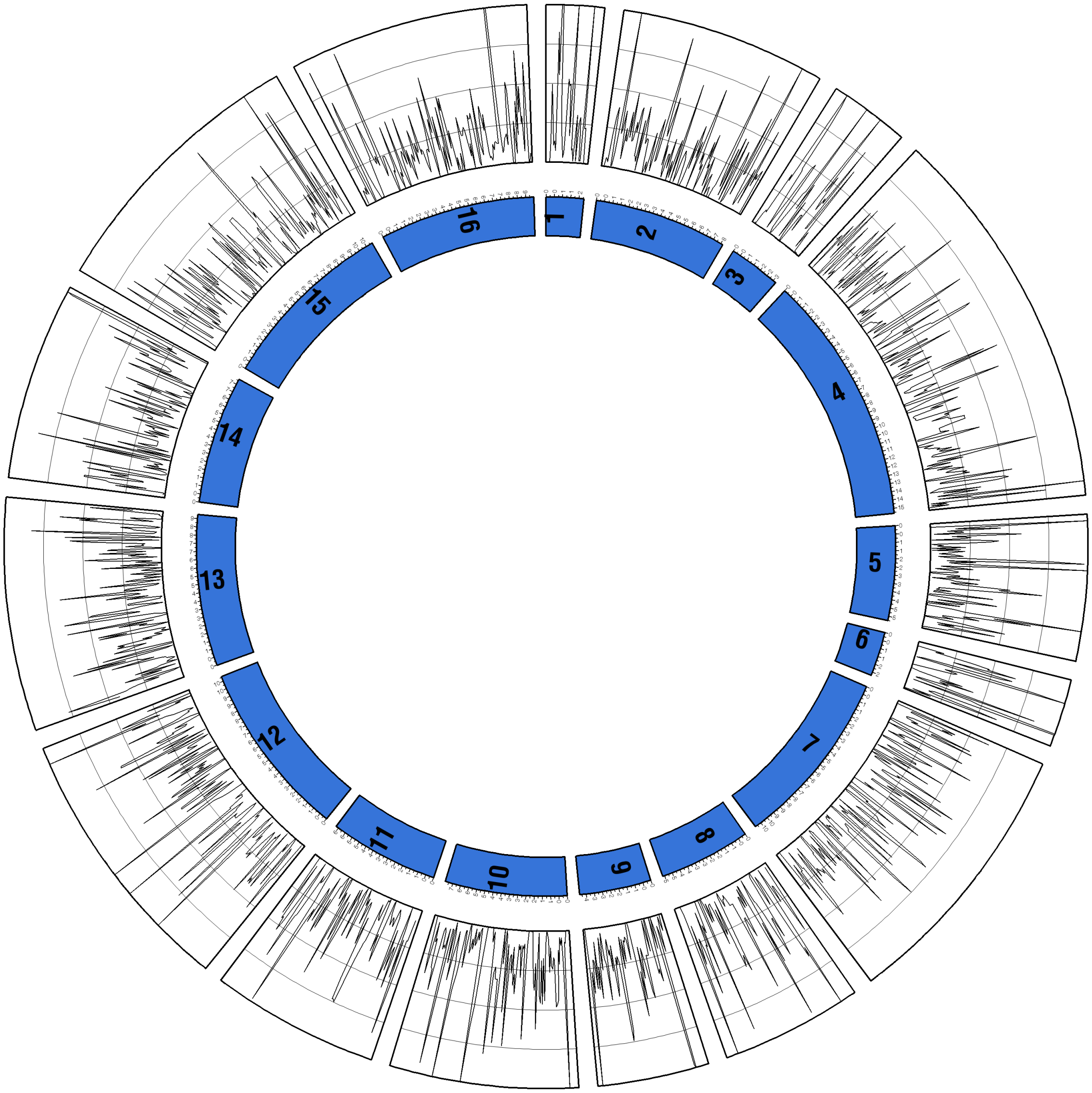

Figure S8. Recombination landscape of the s-way cross at 5kb resolution. The range for the y-axis is from 0 to 2 cM/kb.

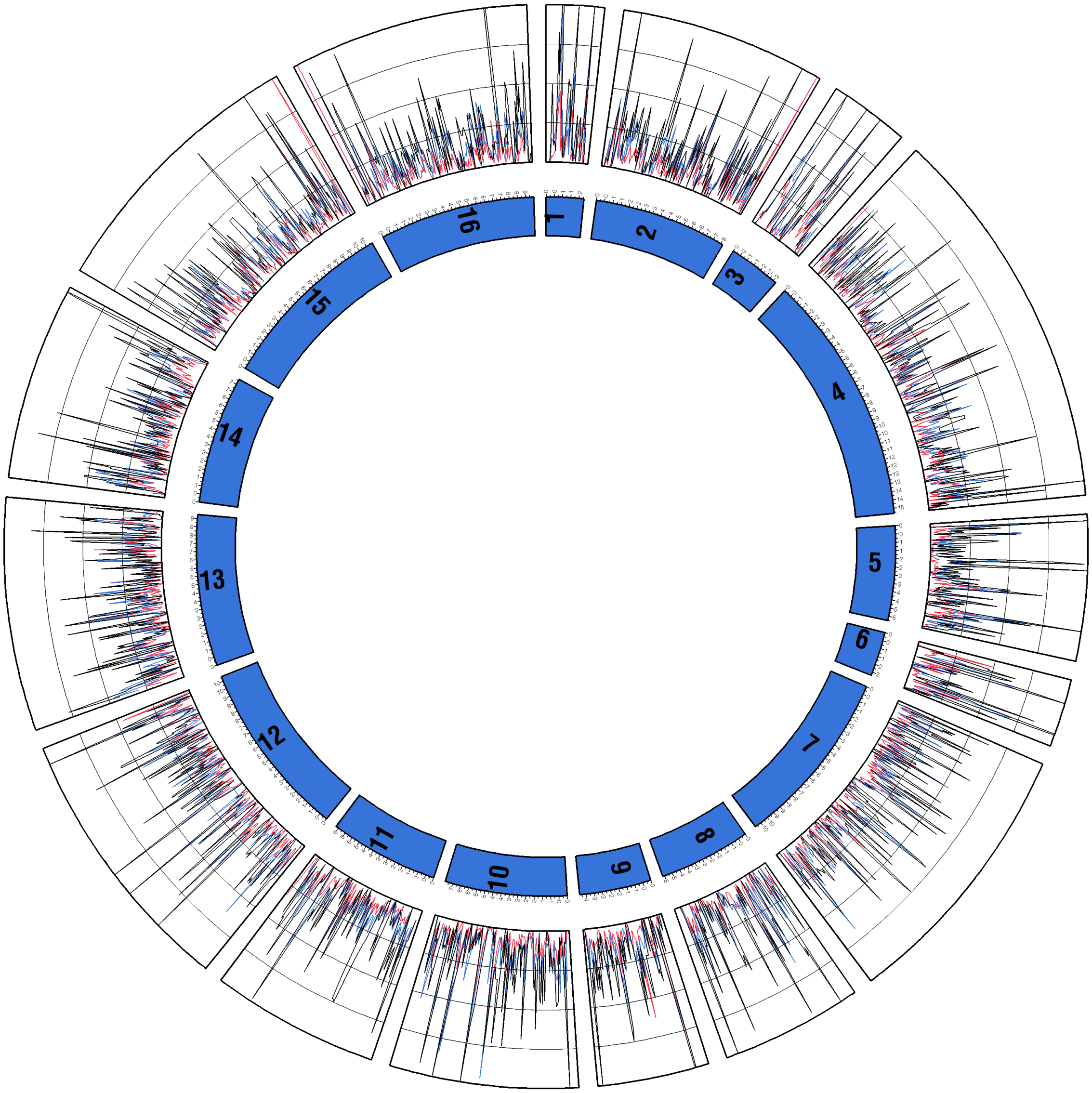

Figure S9. Recombination landscape of the two-way (red), four-way (blue) and s-way (black) crosses at 5kb resolution.
The range for the y-axis is from 0 to 2 cM/kb.
